# IL-32γ attenuates airway fibrosis by modulating the integrin-FAK signaling pathway in fibroblasts

**DOI:** 10.1186/s12931-018-0863-3

**Published:** 2018-09-26

**Authors:** Gyong Hwa Hong, So-Young Park, Hyouk-Soo Kwon, Bo-Ram Bang, Jaechun Lee, Sang-Yeob Kim, Chan-Gi Pack, Soohyun Kim, Keun-Ai Moon, Tae-Bum Kim, Hee-Bom Moon, You Sook Cho

**Affiliations:** 1 Asan Institute for Life Science, Seoul, Korea; 2 Department of Internal Medicine, Division of Allergy and Clinical Immunology, Asan Medical Center, University of Ulsan College of Medicine, 88 Olympic-ro 43-gil, Songpa-gu, Seoul, 138-736 Korea; 3 Department of Internal medicine, Division of Allergy and Respiratory Medicine, Konkuk University Medical Center, Seoul, Korea; 4 Department of Internal Medicine, Jeju National University School of Medicine, Jeju, Korea; 5 Department of Convergence Medicine, University of Ulsan, Seoul, Korea; 6 Laboratory of Cytokine Immunology, Institute of Biomedical Science and Technology, College of Medicine, Konkuk University, Seoul, Korea

**Keywords:** Interleukin-32γ, Asthma, Airway inflammation, Subepithelial fibrosis, Pulmonary fibrosis

## Abstract

**Background:**

Fibrosis in severe asthma often leads to irreversible organ dysfunction. However, the mechanism that regulates fibrosis remains poorly understood. Interleukin (IL)-32 plays a role in several chronic inflammatory diseases, including severe asthma. In this study, we investigated whether IL-32 is involved in fibrosis progression in the lungs.

**Methods:**

Murine models of chronic airway inflammation induced by ovalbumin and *Aspergillus melleus* protease and bleomycin-induced pulmonary fibrosis were employed. We evaluated the degree of tissue fibrosis after treatment with recombinant IL-32γ (rIL-32γ). Expression of fibronectin and α-smooth muscle actin (α-SMA) was examined and the transforming growth factor (TGF)-β-related signaling pathways was evaluated in activated human lung fibroblasts (MRC-5 cells) treated with rIL-32γ.

**Results:**

rIL-32γ significantly attenuated collagen deposition and α-SMA production in both mouse models. rIL-32γ inhibited the production of fibronectin and α-SMA in MRC-5 cells stimulated with TGF-β. Additionally, rIL-32γ suppressed activation of the integrin-FAK-paxillin signaling axis but had no effect on the Smad and non-Smad signaling pathways. rIL-32γ localized outside of MRC-5 cells and inhibited the interaction between integrins and the extracellular matrix without directly binding to intracellular FAK and paxillin.

**Conclusions:**

These results demonstrate that IL-32γ has anti-fibrotic effects and is a novel target for preventing fibrosis.

**Electronic supplementary material:**

The online version of this article (10.1186/s12931-018-0863-3) contains supplementary material, which is available to authorized users.

## Background

Fibrosis, characterized by the accumulation of fibroblasts and excess extracellular matrix, is a common feature of various pathological states in many organs, resulting in dysfunction. Interstitial lung diseases and chronic inflammatory airway diseases of the lungs, such as severe asthma and chronic obstructive pulmonary disease (COPD), lead to sub-bronchial fibrosis and pulmonary fibrosis, both of which result in irreversible structural changes that affect patient survival [1–3]. Because lung fibrosis is mainly a consequence of chronic inflammation, therapeutic strategies have focused on preventing inflammation by administering immunosuppressive agents or anti-inflammatory drugs, including corticosteroids [4, 5]. However, recent studies have suggested that inflammation alone is not sufficient for inducing fibrosis development. Many studies showed that immunosuppressive therapies do not prevent lung fibrosis [6]. To date, targeting fibrosis itself has been unsuccessful.

Interleukin (IL)-32, initially described as NK4 generated by activated T cells or NK cells [7], is produced by various cells, including epithelial cells, endothelial cells, and macrophages. IL-32 induces the production of several pro-inflammatory mediators, such as tumor necrosis factor (TNF)-α, IL-1β, and IL-6, by activating the nuclear factor-κB and p38 mitogen-activated protein kinase signaling pathways [8, 9]. IL-32 is also involved in several chronic inflammatory diseases, such as rheumatoid arthritis and COPD [10–12]. In addition to its role in inflammation, recent studies suggested that IL-32 is involved in liver fibrosis in patients with chronic hepatitis by affecting cytokine induction [13]. Although the precise effects of IL-32 on tissue fibrosis are largely unknown, IL-32 contains an RGD motif, which is known to bind several integrins [14]. Moreover, a 3-dimensional reconstruction model of IL-32 revealed that its structure was highly similar to that of the focal adhesion targeting (FAT) region of focal adhesion kinase (FAK). FAK-related non-kinase, a peptide with a structure similar to the FAT region, inhibits FAK signal transduction [15]. Because the integrin-FAK signaling axis is critical for the development of tissue fibrosis [16, 17], we predicted that IL-32 interrupts the signaling pathway by binding to these molecules, thereby inhibiting FAK activation and alleviating fibrosis.

We hypothesized that IL-32γ modulates fibrosis in chronic airway and lung diseases by disrupting the integrin-FAK signaling pathway. Here, we used murine models of chronic airway inflammation and bleomycin-induced pulmonary fibrosis to examine the role of IL-32γ in fibrosis of the airways and lungs, respectively. We also evaluated the role of IL-32γ in mechanisms underlying fibroblast function.

## Methods

### Generation of murine models of airway inflammation and pulmonary fibrosis

To generate the bleomycin-induced pulmonary fibrosis model, mice were administered intratracheal injection of bleomycin (1 U/kg body weight) on day 2. To evaluate the effect of IL-32γ treatment, mice were administered 500 ng of human recombinant IL-32γ (rIL-32γ) via intranasal injection on days 1, 2, 14, and 28. In this model, rIL-32γ was injected intranasally; 1 h later, bleomycin was injected intratracheally. Mice were sacrificed at 30 days. To generate the chronic asthma model, wild-type (WT) mice were sensitized by intranasal administration of 22 μg of ovalbumin (OVA) and 8 μg of protease (*Aspergillus melleus* protease; Sigma, St. Louis, MO, USA) twice per week for 8 weeks, as previously described [23]. Mice were sacrificed at 58 days. To evaluate the effect of IL-32γ treatment, mice were treated with 500 ng human recombinant IL-32γ (rIL-32γ) 2 h before each immunization. IL-32γ transgenic (TG) mice on a C57BL/6 background were generated as previously described [18]. The Institutional Animal Care and Use Committee approved all experimental procedures (Animal Utilization Protocol 2014-14-013). Additional details are provided in the Additional file 1.

### Histopathologic examination and quantification of tissue fibrosis

Lungs were removed and fixed in 10% neutral-buffered formalin, embedded in paraffin, and sectioned (4 μm). Sections were subjected to Masson’s trichrome staining and immunofluorescence staining using several antibodies. To quantify tissue fibrosis, we measured hydroxyproline levels in the tissue. Additionally, quantification graphs were drawn from intensity measurement data using the Image J program (NIH, Bethesda, MD, USA).

Additional details are provided in the Additional file 1.

### Cell culture and study design

The human lung fibroblast cell line MRC-5 was purchased from the American Type Culture Collection (Manassas, VA, USA). Mouse embryonic fibroblasts (MEFs) obtained from IL-32γ TG mice were also used. MRC-5 cells were seeded at 2 × 10^5^ cells/well and stimulated with recombinant proteins. Expression of various cellular molecules was measured by *Western* blotting, reverse transcription-PCR, and semi-quantitative PCR. Additional details are provided in the Additional file 1. All in vitro experiments were conducted at least 3 times.

### Cell adhesion assay

For crystal violet staining, 96-well culture dishes were coated with collagen (Advanced BioMatrix, Inc., San Diego, CA, USA) and seeded with MRC-5 cells. Plates were incubated for 30, 60, or 180 min. Cells were washed with PBS to remove non-adherent cells, and adhered cells were stained with crystal violet. Additional details are provided in the Additional file 1.

### His pull-down assay and immunoprecipitation

His-tagged IL-32γ was incubated with Ni-NTA agarose beads (Qiagen, Hilden, Germany), which were washed with buffer containing imidazole (Sigma). TGF-β-stimulated MRC-5 cells were lysed and centrifuged; the supernatant was incubated with Ni-NTA agarose-bound IL-32γ. Proteins attached to the beads were subjected to immunoblotting. For immunoprecipitation, MRC-5 cells overexpressing flag-tagged IL-32γ were lysed and incubated with protein G Sepharose beads (GE Healthcare, Little Chalfont, UK) coated with an anti-flag antibody. Proteins were electrotransferred for immunoblotting. Additional details are provided in the Additional file 1.

### Live cell imaging of IL-32γ

MRC-5 cells were cultured in a μ-Dish 35 mm, High, IbiTreat (Ibidi GmbH, Martinsried, Germany) and treated with Flamma496-labeled IL-32γ. After 10 min, the cells were washed with medium. Fluorescence images were obtained under a Nikon Ti-E inverted I wamicroscope (Tokyo, Japan) equipped with PFS, iXon Ultra 897 EMCCD camera (Andor Technology, Belfast, UK), and excitation and emission filter wheels.

### Statistical analysis

All data are reported as the mean ± standard error of mean. Means were compared using the Mann–Whitney test in GraphPad Prism software (version 4.0; GraphPad, Inc., La Jolla, CA, USA). A value of *p* < 0.05 was considered statistically significant.

## Results

### IL-32γ modulates fibrosis in mouse models of airway inflammation and pulmonary fibrosis

**Fig. 1:**
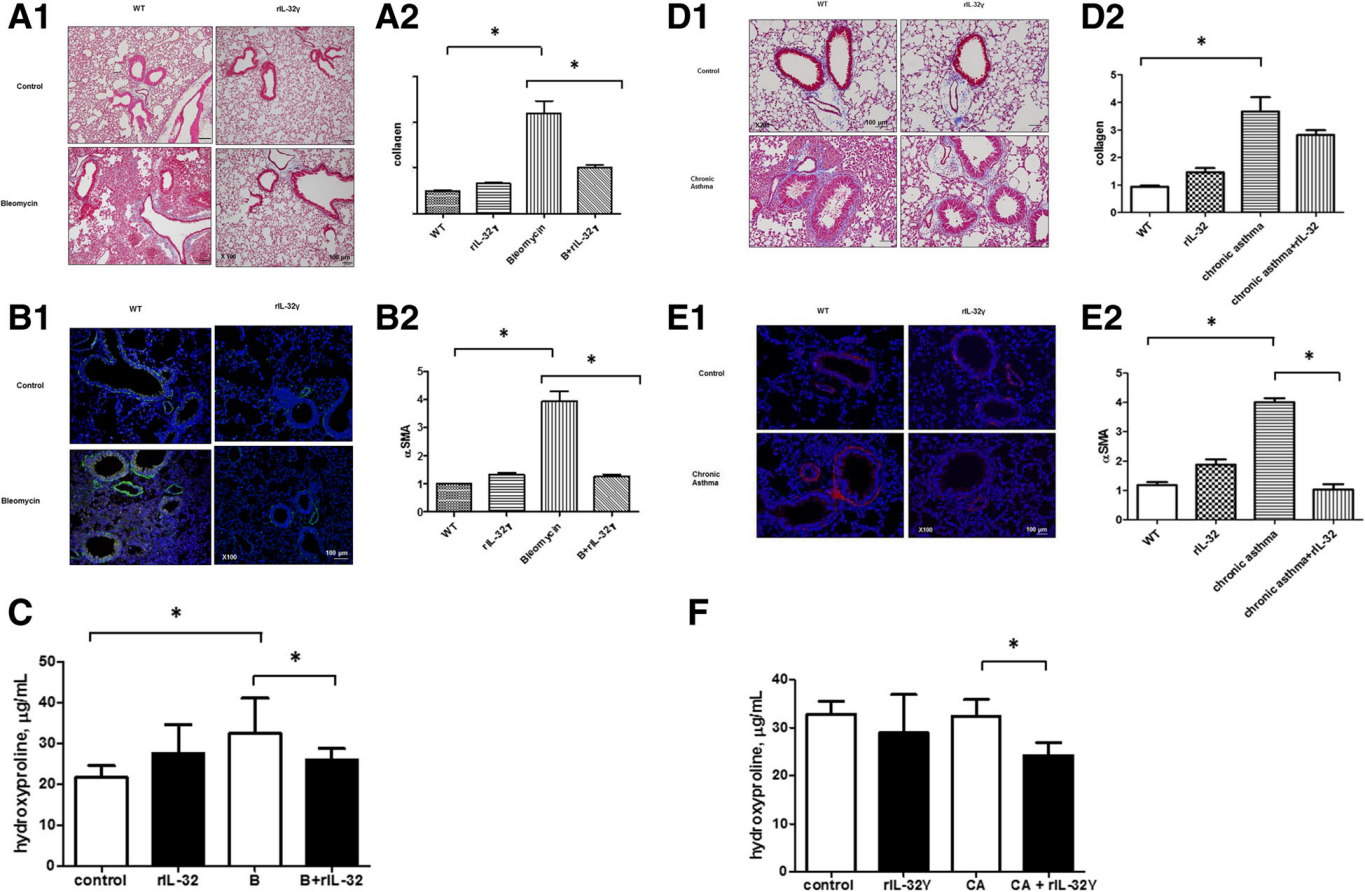
Human IL-32γ prevents fibrosis in chronic asthma and bleomycin-induced pulmonary fibrosis models. **a** Evaluation of collagen deposits in the lungs of bleomycin-induced mice using Masson’s trichrome stain (original magnification: 100×). The quantification graphs of histological analysis in bleomycin-induced fibrosis groups. **b** Immunofluorescence analysis of α-SMA (green) expression in the lungs of bleomycin-induced mice. DAPI staining is blue (original magnification: 100×). **c** Hydroxyproline quantification. In the group with bleomycin-induced fibrosis treated with rIL-32γ (*N* = 5, B ± rIL-32γ), hydroxyproline levels tended to decrease compared to in the non-rIL-32γ-treated bleomycin-induced fibrosis model (*N* = 6, B) (32.40 ± 3.885 vs. 26.70 ± 1.287, *P* = 0.166). **d** Evaluation of collagen deposition in the lungs of chronic asthmatic mice using Masson’s trichrome stain (original magnification: × 200). **e** Immunofluorescence analysis of α-SMA (red) expression in the lungs of mice with chronic asthma. DAPI staining is blue (original magnification: × 200). **f** Hydroxyproline quantification graph. Similar results were obtained in each independent experiment, each using five mice per group (32.35 ± 1.752 vs. 24.20 ± 1.344, *P* = 0.010). *P ≤ 0.05

First, histopathological analysis of bleomycin-induced lung fibrosis was conducted to determine the effect of IL-32γ on pulmonary fibrosis. Treatment with rIL-32γ significantly reduced collagen deposition and α-smooth muscle actin (SMA) expression (Fig. 1a and b). Hydroxyproline levels showed a tendency to be lower in the bleomycin-induced fibrosis group treated with rIL-32γ than in the group without rIL-32γ treatment (34.01 ± 7.24 vs. 25.52 ± 3.66, *p* = 0.048; Fig. 1c). Next, to determine the effect of IL-32γ on airway remodeling in chronic asthma, a murine model of chronic airway inflammation with subepithelial fibrosis was treated with rIL-32γ. Treatment with rIL-32γ reduced peribronchial collagen deposition (Fig. 1d). This was accompanied by reduced expression of α-SMA, a marker of activated fibroblasts, around the bronchi of treated mice (Fig. 1e). Figure 1f is a graph showing quantification of hydroxyproline. Hydroxyproline levels were significantly lower in the chronic asthma model treated with rIL-32γ **(**32.35 ± 1.752 vs. 24.20 ± 1.344, *P* = 0.010).

### rIL-32γ attenuates fibroblast activation

**Fig. 2:**
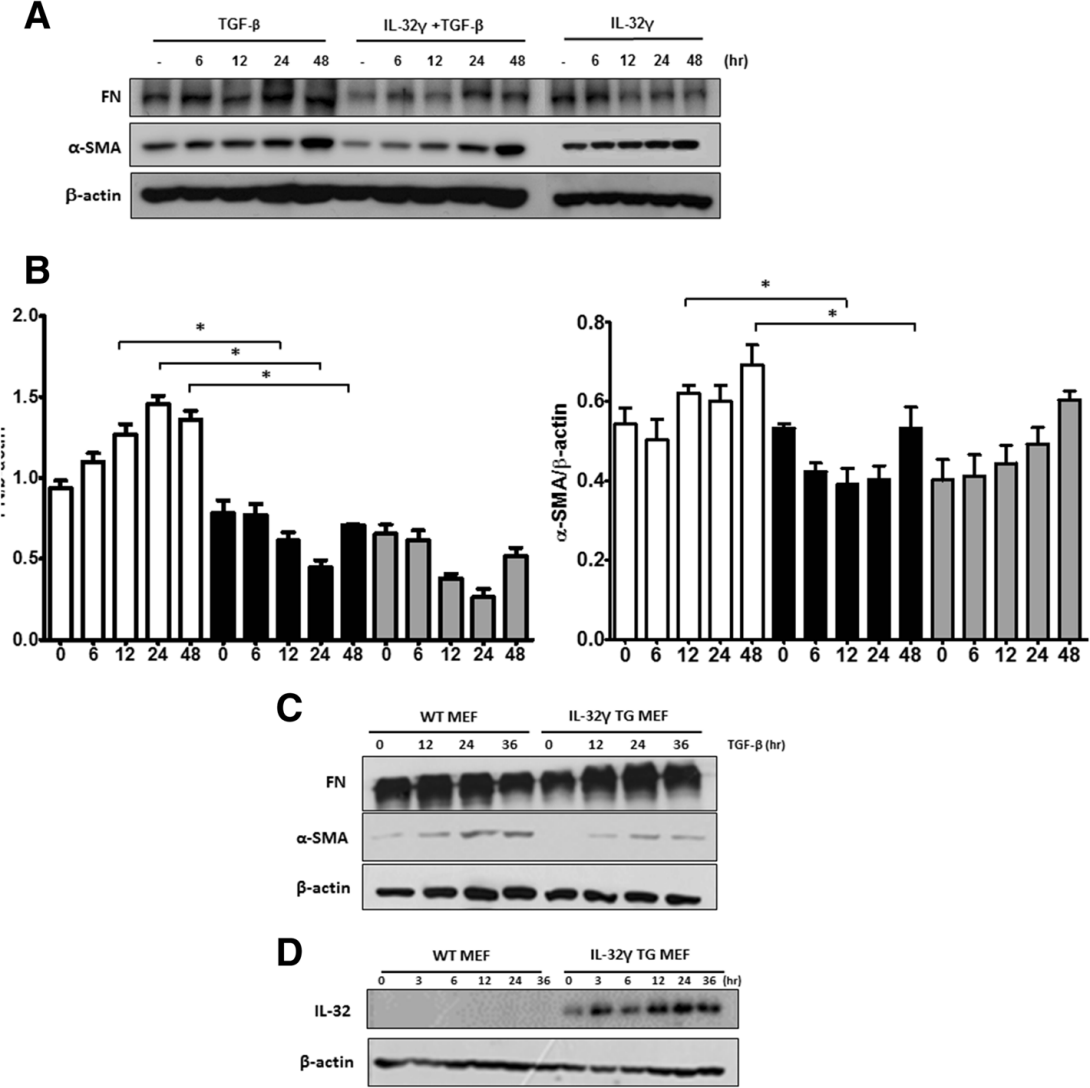
Exogenous, but not endogenous, IL-32γ attenuates fibroblast activation. Fibronectin and α-SMA expression was detected in rIL-32γ (150 ng/mL)-pretreated MRC-5 cells after TGF-β (5 ng/mL) stimulation. **a**. Quantification graphs is shown (**b**). Fibronectin and α-SMA expression are shown in IL-32γ-expressing MEFs after TGF-β (5 ng/mL) stimulation (**c**). Endogenous IL-32 expression (**d**). Data are representative of three independent experiments

Next, to determine whether IL-32γ affects fibrosis by regulating fibroblast activation, expression of fibronectin and α-SMA was measured in the human fibroblast cell line MRC-5 after treatment with TGF-β in the presence or absence of rIL-32γ. Fibronectin expression in rIL-32γ-treated cells was significantly lower than that in untreated cells, whereas α-SMA expression was slightly lower at early time points (Fig. 2a and b). However, overexpression of endogenous intracellular IL-32γ did not noticeably affect the production of fibronectin and α-SMA by MEFs from WT or IL-32γ TG mice (Fig. 2c and Additional file 2: Figure S1). Endogenous IL-32 expression is shown in Fig. 2d.

### Anti-fibrotic effect of rIL-32γ occurs independently of TNF-α

**Fig. 3:**
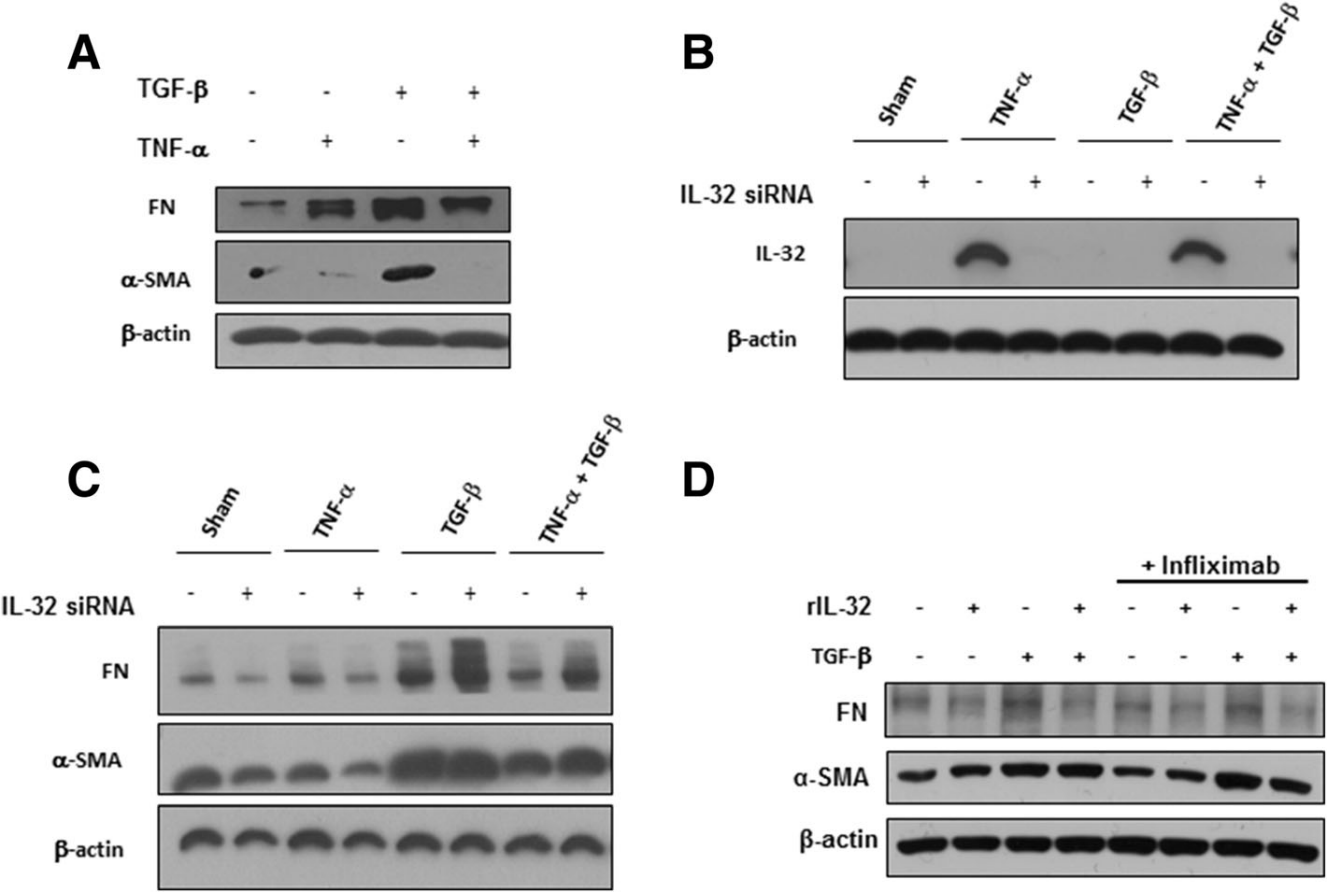
Anti-fibrotic effects of rIL-32γ are independent of TNF-α. **a** Fibronectin and α-SMA expression in MRC-5 cells after 24 h of stimulation with TNF-α (10 ng/mL) and TGF-β (5 ng/mL). **b** MRC-5 cells were transfected with IL-32 siRNA and then stimulated with TNF-α (10 ng/mL) or TGF-β (5 ng/mL). **c** Fibronectin and α-SMA expression in cell lysates was detected. **d** Infliximab-pretreated MRC-5 cells were stimulated with IL-32γ (150 ng/mL) and TGF-β (5 ng/mL), and fibronectin and α-SMA expression in the cell lysate was detected. Results are representative of two independent experiments, each showing similar results

Because IL-32 induces the production of TNF-α and vice versa, we next examined whether IL-32γ exerts anti-fibrotic effects by inducing TNF-α expression. First, we found that significant expression of IL-32γ mRNA was induced by TNF-α, although no significant change in TNF-α mRNA expression was observed (see Additional file 3: Figure S2A and B). Similar to IL-32γ, treatment with rTNF-α inhibited the expression of fibronectin and α-SMA in TGF-β-stimulated MRC-5 cells (Fig. 3a). However, IL-32 was not expressed by rTNF-α under IL-32γ-knockdown conditions (Fig. 3b) and an anti-fibrotic effect of TNF-α was not observed in IL-32γ-knockdown MRC-5 cells (Fig. 3c and Additional file 4: Figure S3A). Additionally, rIL-32γ inhibited fibronectin and α-SMA expression after TNF-α inhibitor treatment (Fig. 3d and Additional file 4: Figure S3B).

### rIL-32γ does not appear to be involved in TGF-β-mediated Smad or non-Smad signaling

**Fig. 4:**
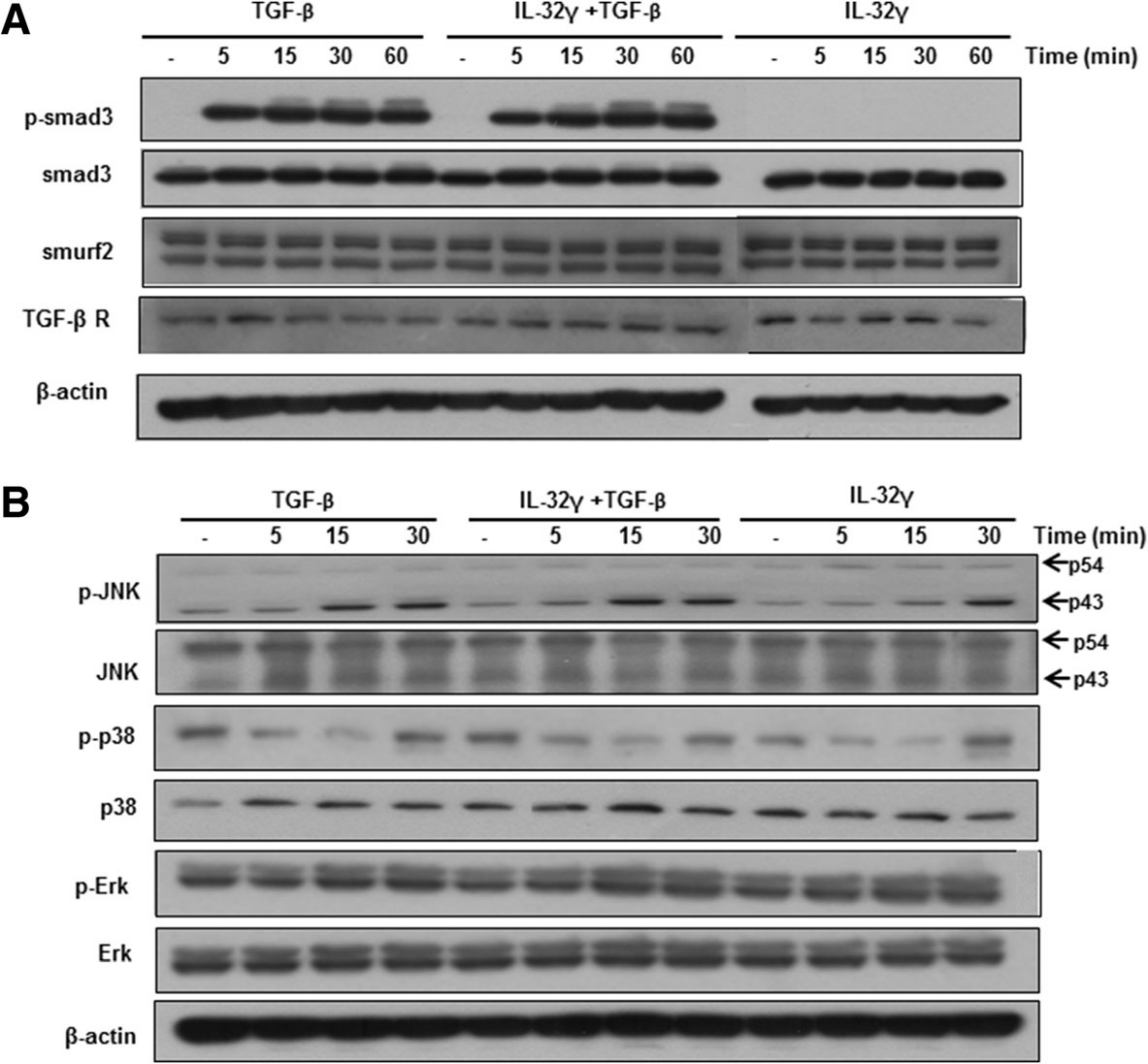
rIL-32γ has no effect on TGF-β-mediated Smad or non-Smad signaling pathways. MRC-5 wells were stimulated with TGF-β (5 ng/mL) in the presence or absence of rIL-32γ and then harvested at the indicated times. Western blot analysis was performed to examine the expression of proteins in the Smad signaling (**a**) and non-Smad signaling pathways (**b**). Results are representative of three independent experiments

We next examined the effect of IL-32γ on activation of the Smad pathway, a well-known TGF-β-mediated signaling pathway. There were no significant differences in the expression of Smad signaling molecules (p-Smad 3, smuf2, and TGF-β receptor 1), regardless of rIL-32γ treatment (Fig. 4a). Next, we examined whether the non-Smad pathway plays a role in the anti-fibrotic effects of rIL-32γ. We found no significant differences in JNK, Erk, and p38 activation between MRC cells treated with rIL-32γ and untreated cells (Fig. 4b).

### rIL-32γ inhibits integrin-mediated FAK/paxillin activation

**Fig. 5:**
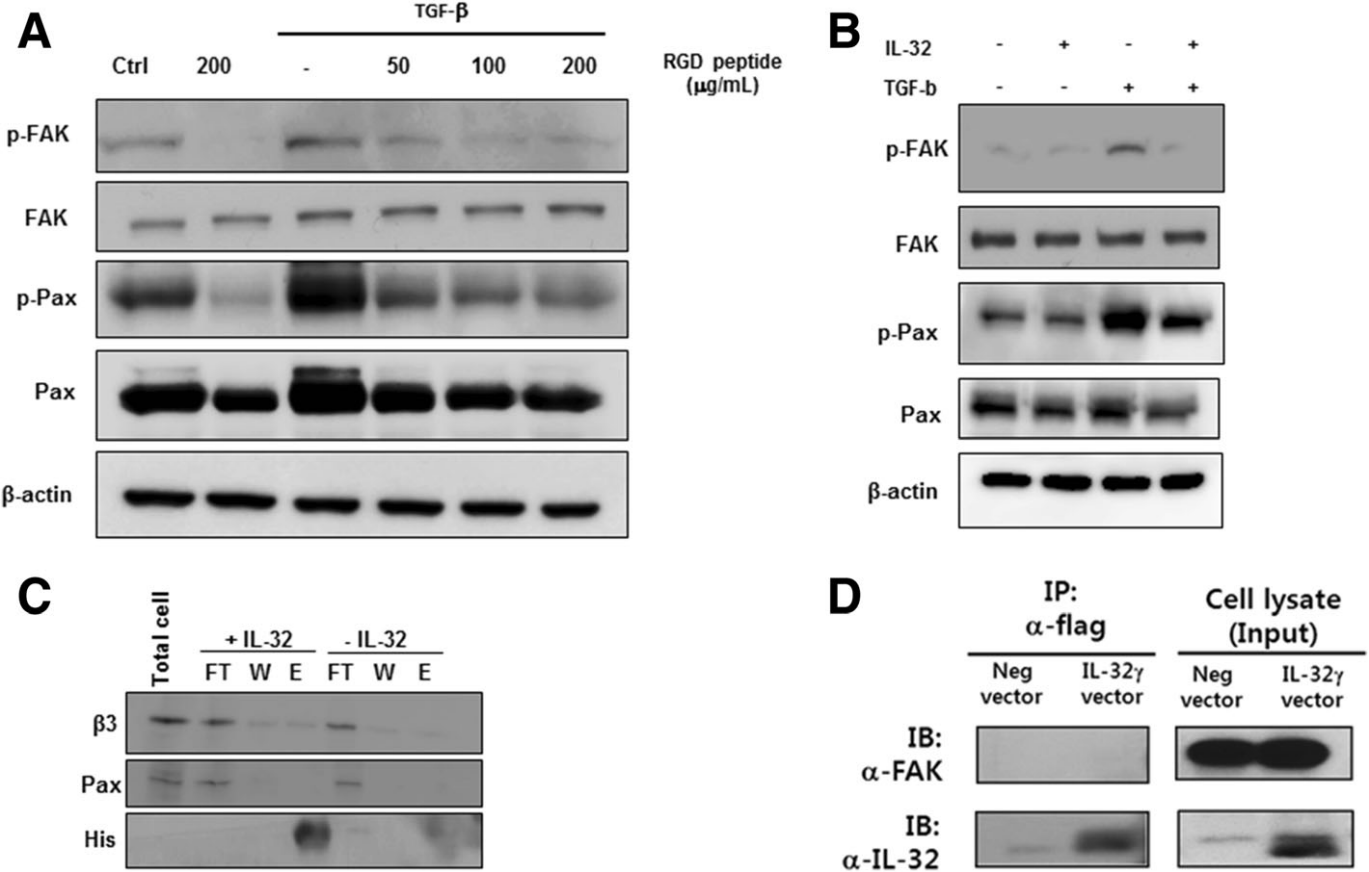
rIL-32γ inhibits integrin-mediated activation of FAK/paxillin. Phosphorylation of FAK and paxillin was detected in TGF-β (5 ng/mL)-stimulated MRC-5 cells pretreated with an RGD peptide (**a**) or rIL-32γ (**b**). Activated FAK and paxillin were detected after 24 h. Results are representative of three independent experiments. **c** MRC-5 cells were stimulated with TGF-β for 24 h, and His-tagged rIL-32γ was precipitated from cell lysates using Ni-NTA beads. Bound proteins were analyzed by *Western* blotting with antibodies specific for integrin β3, paxillin, and the His-tag. **d** Flag-tagged IL-32γ-overexpressing MRC-5 cells were stimulated with TGF-β and harvested at 24 h. Flag-tagged IL-32γ was then immunoprecipitated from cell lysates using an anti-flag antibody followed by immunoblotting with an anti-FAK antibody. Similar results were obtained from two independent experiments. FT, flow-through; W, wash; E, elution

Next, we examined integrin-dependent activation of FAK and paxillin, a critical pathway in fibroblast activation, after treatment with the RGD tripeptide and integrin blocker. RGD peptide inhibited signaling by both FAK and paxillin in MRC-5 cells stimulated with TGF-β (Fig. 5a). Interestingly, rIL-32γ inhibited FAK and paxillin signaling in a manner similar to that of RGD peptide (Fig. 5b).

To investigate how IL-32γ regulates the integrin-FAK-paxillin signaling pathway, we performed a protein-protein binding assay to determine whether IL-32 directly binds to integrin β3, paxillin, or FAK. Both integrin β3 and paxillin were detected in the total cell lysate and flow-through lanes, but no bands were detected in the wash and elution fractions (Fig. 5c). This suggests that these proteins do not directly bind to IL-32γ. Additionally, an anti-flag-IL-32γ antibody did not immunoprecipitate with FAK (Fig. 5d).

### rIL-32γ is localized on the cell surface

**Fig. 6:**
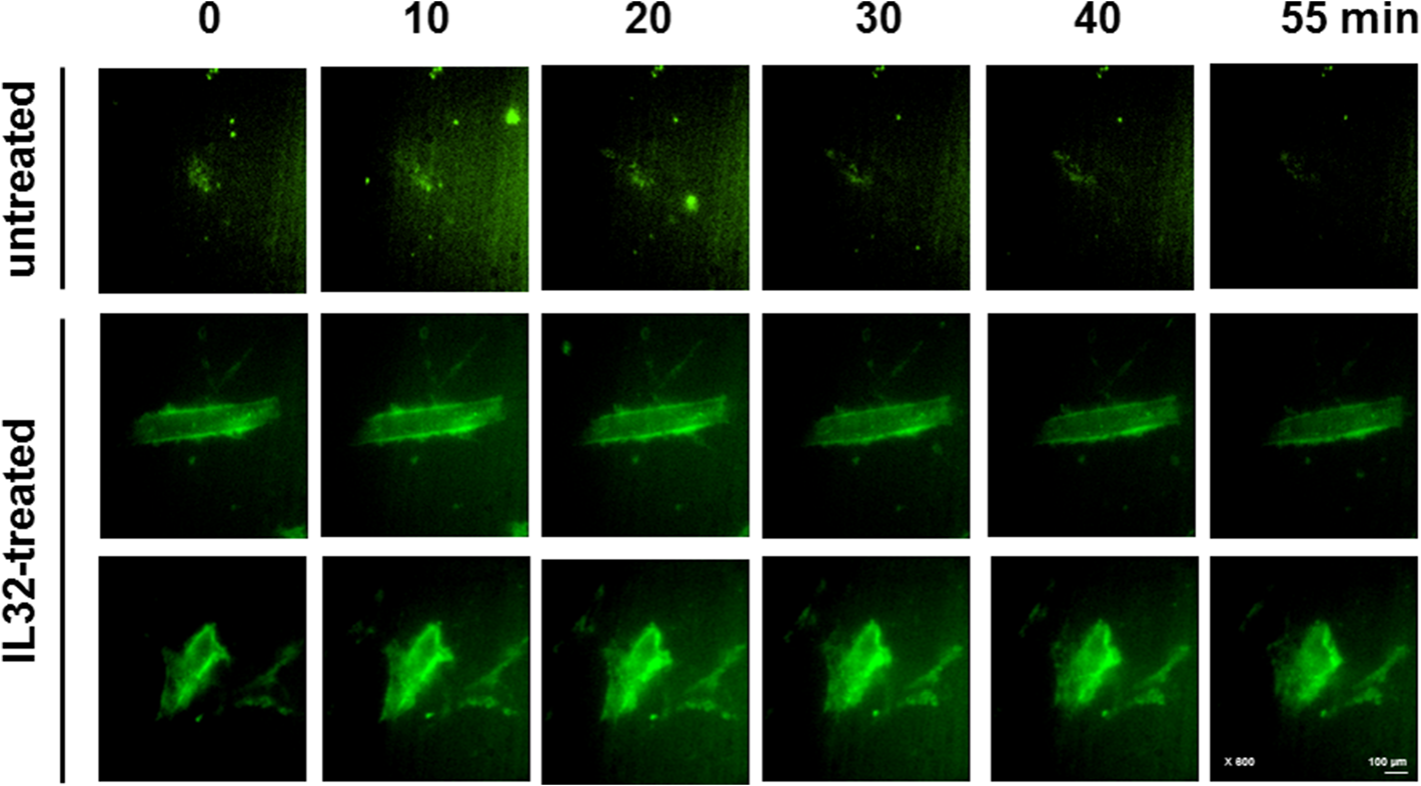
rIL-32γ localizes extracellularly. Live cell imaging of MRC-5 cells at 10–60 min post-incubation with Flamma496-labeled IL-32γ (magnification, 600×; green color)


To determine the mechanism by which rIL-32γ inhibits activation of the FAK/paxillin pathway, we next examined the location of rIL-32γ by live cell imaging for 60 min. rIL-32γ was located outside of MRC-5 cells after 60 min, suggesting that it does not enter cells by endocytosis and is not degraded; therefore, IL-32γ acts extracellularly, at least during the period examined (Fig. 6, see Additional files 5 and 6: Video 1 and 2 in the online Supplement).

**Additional file 5:** **Supplementary video.** (MP4 2.42 mb)
**Additional file 6:** **Supplementary video.** (MP4 1.98 mb)


### rIL-32γ modulates the interaction between integrins and the extracellular matrix

**Fig. 7:**
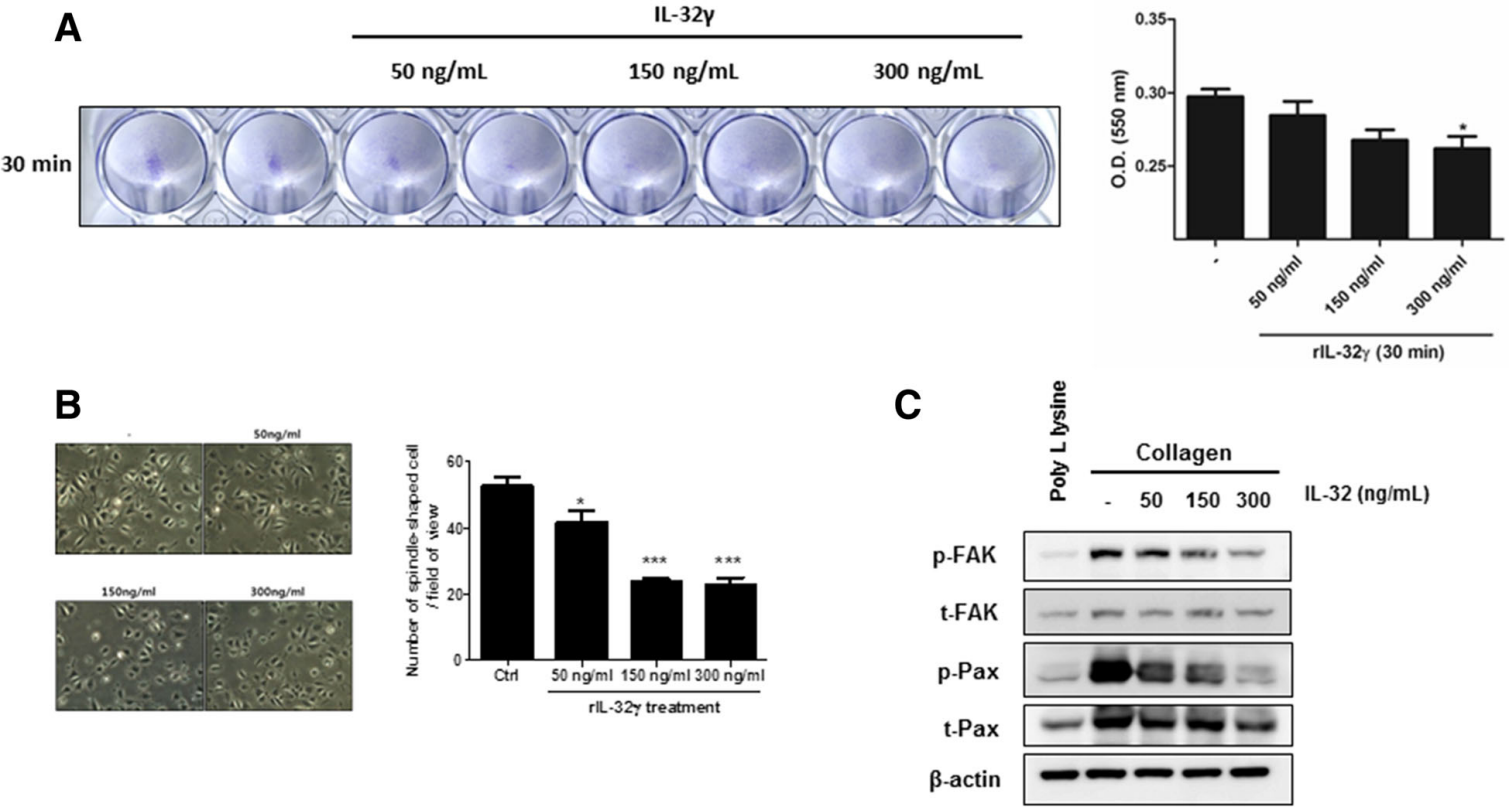
rIL-32γ modulates the interaction between integrins and the extracellular matrix. MRC-5 cells were plated on collagen-coated plates in the presence/absence of rIL-32γ. **a** Adherent MRC-5 cells were stained with crystal violet immediately after the adhesion assay (left) and optical density values from the dissolved crystals are shown (right). Similar results were obtained from three independent experiments. *P < 0.05 (**b**) Adherent cells were observed at 30 min under a microscope (original magnification: 100×). Similar results were obtained from two independent experiments. *P < 0.05, ***P < 0.0001 (**c**) Phosphorylation of FAK and paxillin was detected after MRC-5 cells attached to collagen-coated plates for 24 h in the presence/absence of rIL-32γ. Similar results were obtained from two independent experiments

To examine the effect of IL-32γ on integrin signaling, we examined the adhesion of MRC-5 cells to collagen-coated plates in the presence/absence of rIL-32γ. MRC-5 cells adhered to collagen within 30 min in the absence of rIL-32γ; however, the process was impeded in the presence of rIL-32γ (Fig. 7a). Moreover, the number of spindle-shaped MRC-5 cells was much lower in the presence of rIL-32γ, even after 30 min (Fig. 7b). Interestingly, rIL-32γ suppressed integrin/collagen-mediated activation of FAK and paxillin, which is typically induced by cell adhesion to collagen-coated plates in the absence of any other stimulation (Fig. 7c). Finally, we examined the effect of IL-32γ on integrin expression in MRC-5 cells, as TGF-β upregulates integrin expression. Semi-quantitative PCR revealed increased expression of integrin β3 and reduced expression of integrin β8 following TGF-β stimulation. This pattern was not altered by IL-32γ treatment (see Additional file 7: Figure S4).

## Discussion

This study demonstrated the anti-fibrotic effect of IL-32γ both in vitro and in vivo. We showed that rIL-32γ regulates fibroblast activation by modulating the integrin-FAK signaling pathway. Thus, rIL-32γ may be useful for inhibiting tissue fibrosis in the clinical setting.

The mechanism of tissue fibrosis is closely related to that of wound repair, which is a normal healing process in injured tissues. However, dysregulated fibrosis can lead to severe organ dysfunction, which is typically irreversible and has a fatal outcome in many disease states. In the lungs, for example, progressive parenchymal fibrosis is a consequence of serious pulmonary fibrotic diseases such as idiopathic pulmonary fibrosis, leading to high mortality. Additionally, bronchial subepithelial fibrosis can cause irreversible fixed airway obstruction, as observed in chronic inflammatory airway diseases such as chronic severe asthma and COPD, which can become critical if untreated.

Although lung fibrogenesis is thought to result from chronic inflammation, numerous studies have suggested that fibrosis is not completely dependent on inflammatory processes and that anti-inflammatory therapeutic strategies are not always effective. Thus, therapeutic trials have shifted their focus from anti-inflammatory targets to anti-fibrotic targets, as many studies demonstrated that such mechanisms underlie the development of fibrosis [19–22]. However, therapeutic agents that effectively control fibrosis are lacking; therefore, there is an urgent need to identify novel molecules with potent anti-fibrotic activities.

IL-32, previously considered a pro-inflammatory cytokine, is a multifunctional protein with a potential role in lung diseases [12, 23–25]. We previously showed that IL-32γ modulates immune responses by recruiting IL-10-producing monocytic cells in a chronic asthma model [24]. Here, we observed that IL-32γ also exhibits a strong anti-fibrotic effect in a model of sub-bronchial fibrosis. Because chronic inflammation is a major factor driving the progression of fibrosis, its apparent suppressive effect on airway fibrosis may be completely dependent on the anti-inflammatory effects of IL-32γ. Thus, we examined the modulatory effects of IL-32γ in a bleomycin-induced lung injury model, which is considered a prototype of tissue fibrosis but displays lower accumulation of immune cells in the lungs. This is of interest because IL-32γ is a putative immunomodulatory cytokine. The results of the current study suggest that IL-32γ has a novel function in lung fibrosis, as well as anti-inflammatory effects on chronic airway inflammation.

We also used human fibroblasts to further investigate the mechanism underlying the anti-fibrotic effect of IL-32γ, as excessive accumulation of extracellular matrix produced by activated fibroblasts is a major pathological feature in tissue fibrosis, and any possible effects of inflammation in an animal can be excluded. MRC-5 cells were stimulated with TGF-β, which induces fibroblasts to differentiate into fibronectin- and α-SMA-expressing myofibroblasts. We found that IL-32γ effectively inhibited expression of these activation markers upon TGF-β stimulation. Previous studies showed that TNF-α and IL-32γ induce one another. Additionally, TNF-α inhibits the TGF-β-induced Smad signaling pathway [26–29]. Thus, we used cells in which IL-32γ had been silenced and a TNF-α-blocking agent to determine the exact mechanism underlying the suppressive effect of IL-32γ on fibroblast activation. Furthermore, the intracellular pathways linked to the Smad and non-Smad signaling pathways were assessed. We found that the mechanism underlying the role of IL-32γ in fibrogenesis was not dependent on TNF-α expression, nor was it associated with activation of TGF-β downstream of the Smad or non-Smad signaling pathways.

Previous studies indicated that TGF-β-induced fibroblast activation depends on the integrin signaling pathway through FAK/paxillin activation [16, 30–32]. Protein structure modeling suggested that IL-32γ is involved in integrin activation and downstream signaling pathways [14, 33]. In fact, IL-32γ contains an RGD motif that binds to integrins; indeed, several isoforms of IL-32 bind to integrin αVβ3. In addition, IL-32 has a structure resembling the FAT region of FAK (similar to an FAK-inhibitory peptide). However, these studies examined only IL-32α and β, although IL-32γ is considered the most active form [34].

We found that rIL-32γ inhibited the phosphorylation of FAK and paxillin in TGF-β-stimulated fibroblasts without directly binding to these molecules. Based on these results, extracellular rIL-32γ regulates TGF-β-mediated fibroblast activation without entering the cell. Indeed, we observed that rIL-32γ treatment inhibited integrin-mediated cell adhesion, although rIL-32γ remained outside the cell. These results strongly suggest that IL-32γ is involved in the development of tissue fibrosis, likely by disrupting the binding between integrins expressed in the cellular membrane and the extracellular matrix.

No study has fully identified an IL-32-associated pathway in the context of fibrosis, raising the question of whether IL-32 is released by dead cells or via a specific secretory pathway. Notably, in the early phase of several diseases, IL-32 is produced by activated T cells, monocytes, and NK cells and acts as a pro-inflammatory cytokine that stimulates TNF-α, IL-6, and IL-8 production [8, 12, 35, 36]. Because recent studies showed that IL-32 is not secreted [24, 37], IL-32γ released from injured epithelial cells in patients with chronic inflammatory diseases, including those with mycobacterium avium complex pulmonary disease and idiopathic inflammatory bowel disease [22, 37], may play a regulatory role in inflammation or tissue remodeling. For instance, our previous study showed that rIL-32γ suppresses chronic airway inflammation, which is closely associated with airway remodeling [24].

There were some limitations to the current study. First, to obtain more convincing and direct evidence to evaluate our hypothesis, mutations or deletions of the RGD motif of IL-32γ should be used. Second, our results do not clearly define the precise function of intracellular and extracellular IL-32γ. Further studies are necessary to resolve these questions.

**Fig. 8:**
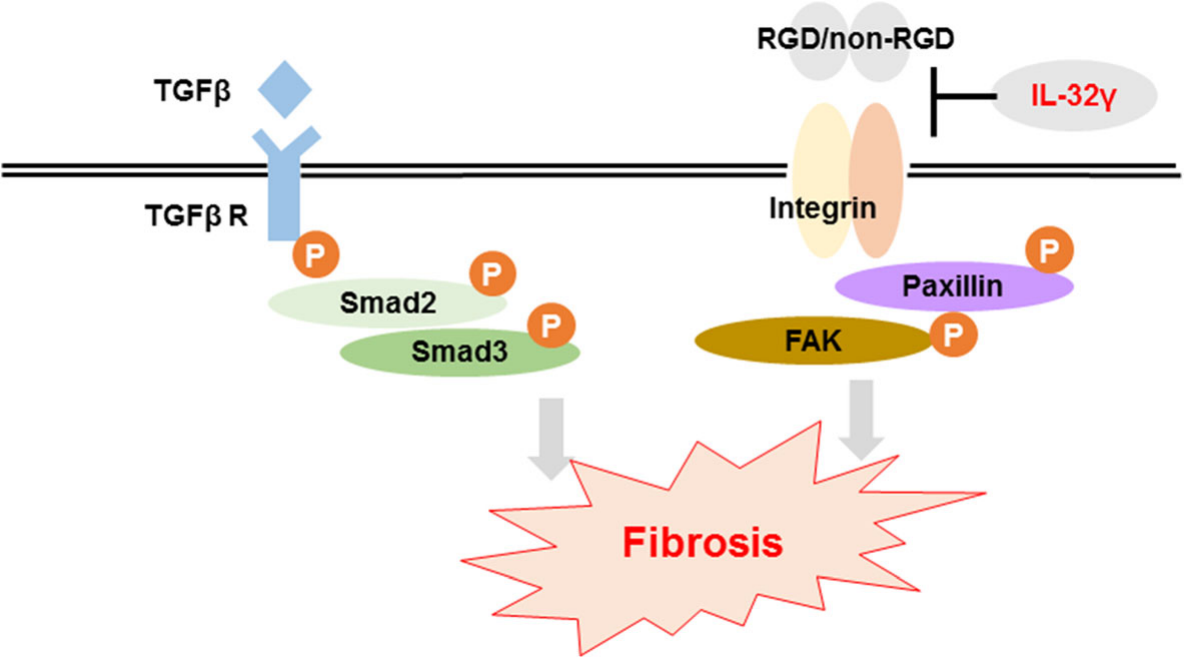
Suggested role of IL-32γ in the fibrosis pathway. Extracellular IL-32γ suppresses activation of the integrin-FAK-paxillin signaling pathway to exert anti-fibrotic effects but has no effect on the TGF β-Smad signaling pathway

In summary, IL-32γ has anti-fibrotic effects likely by blocking the integrin-FAK-paxillin pathway **(**Fig. 8). Therefore, administration of rIL-32γ may play a pivotal role in modulating both inflammation and fibrosis in patients in which inflammation-related fibrosis pathways are activated.

## Conclusions

The present study suggested that IL-32γ prevents tissue damage by regulating fibroblast activation in the chronic stage. The mechanism underlying this modulatory effect may involve disruption of integrin/FAK signaling cascades, without the need for IL-32γ to directly bind molecules involved in these cascades. Thus, IL-32γ is a new candidate for the treatment of lung fibrosis.

## Additional files

Additional file 1: Additional methods detail. (DOC 46 kb)

Additional file 2: **Figure S1.** Extracelular IL-32γ suppresses fibroblast activation. Endogenous IL-32γ did not significantly suppress the expression of fibronectin and α-SMA. (TIF 202 kb)

Additional file 3: **Figure S2.** IL-32γ mRNA expression was induced by TNF-α. MRC-5 cells were stimulated with each cytokine including LPS (1 μg/mL), Poly I: C (10 μg/mL), TNF-α (10 ng/mL), IL-32γ (150 ng/mL), TGF-β (5 ng/mL), and IL-1β (10 ng/mL). After 24-h stimulation, mRNA level of IL-32γ (A) and TNF-α (B) were measured by quantitative PCR. (TIF 71 kb)

Additional file 4: **Figure S3.** Anti-fibrotic effect of rIL-32γ is independent of TNF-α. Anti-fibrotic effect of TNF-α was not observed in IL-32γ-knockdown MRC-5 cells (A). rIL-32γ suppressed the expression of fibronectin and α-SMA after TNF-α inhibitor treatment (B). (TIF 96 kb)

Additional file 7: **Figure S4.** Integrin expression in activated fibroblast is not affected by rIL-32γ. The integrin mRNA levels of α2, αv, β1, β3, β5, β8, and the GAPDH mRNA level were determined by semi-quantitative PCR in MRC-5 after TGF-β or rIL-32γ treatments. (TIF 111 kb)

## Abbreviations

COPD: Chronic obstructive pulmonary disease
FAK: Focal adhesion kinase
FAT: Focal adhesion targeting
IL-32: Interleukin 32
MEFs: Mouse embryonic fibroblasts
OVA: Ovalbumin
rIL-32γ: Recombinant interleukin 32 gamma
TG: Transgenic
TGF: Transforming growth factor
TNF: Tumor necrosis factor
WT: Wild-type
α-SMA: Alpha smooth muscle actin
