# Supplementary material for: IL-32γ attenuates airway fibrosis by modulating the integrin-FAK signaling pathway in fibroblasts
Source: Respir Res. 2018 Sep 26;19:188. doi: 10.1186/s12931-018-0863-3 (PMC6158920; doi:10.1186/s12931-018-0863-3)
Supplement: Supplementary file 3 — Additional methods detail. (DOC 46 kb) [file 12931_2018_863_MOESM1_ESM.doc]

**Methods**

***Generation of murine models of airway inflammation and pulmonary fibrosis***

All mice were bred and maintained in a specific pathogen-free animal facility. IL-32γ transgenic mice on a C57BL/6 background were generated as previously described . Briefly, the open reading frame of IL-32γ cDNA was transferred into pCAGGS. The entire sequence was linearized with *Sal*I and microinjected into mouse zygotes. Transgenic mice showed no physical abnormalities and were screened by RT-PCR. Wild-type (WT) C57BL/6 and Balb/c mice were purchased from OrientBio (Gapyong, Gyeonggi-do, Korea) and used as a control. Bronchoalveolar lavage fluid (BALF) and lung tissues were obtained from mice 24 h after the last immunization for further analysis. To generate the bleomycin-induced pulmonary fibrosis model, 6-week-old female C57BL/6 WT mice were injected with bleomycin intratracheally (1 U/kg body weight). To establish the chronic asthma model, 6-week-old female C57BL/6 mice were immunized with ovalbumin and protease (*Aspergillus melleus* protease, Sigma, St. Louis, MO, USA) twice per week for 8 weeks via the intranasal route. IL-32γ transgenic mice on a C57BL/6 background were generated as described.

Animal study protocols were approved by the Institutional Animal Care and Use Committee of Asan Medical Center, Korea.

***Histopathology and quantification of tissue fibrosis***

Lungs collected from mice (N = 5) in each treatment group were perfused with 5 mL of phosphate-buffered saline (PBS) through the right ventricle. The lung was inflated by intratracheal infusion of 0.3% low-melting agar at 25 cm H_2_O. The inflated lung was fixed with 10% neutral-buffered formalin. Fixed lungs were embedded in paraffin and sectioned at 4 μm. Sections were deparaffinized, rehydrated, subjected to antigen retrieval by boiling in 10 mM sodium citrate buffer, pH 6.0, for 15 min, and blocked in 3% H_2_O_2_ (DaKo Peroxidase Blocking Solution, Carpinteria, CA, USA) for 10 min at room temperature in a humidity chamber. To examine collagen deposition, lung sections were stained with Masson’s trichrome. The slides were washed 3 times with PBS, blocked with 0.25% casein in PBS (DaKo Protein Block Serum-Free) for 15 min at room temperature in a humid chamber, and then incubated with antibodies against α-SMA (Cell Signaling, Inc., Danvers, MA, USA) overnight in the dark at 4°C. After washing 3 times with PBS, the slides were incubated with fluorescein isothiocyanate-conjugated goat anti-rabbit IgG and cy3-donkeyAnti-goat IgG for 1 h at room temperature in the dark and then detected using a DaKo EnVision HRP/DAB system (Dako). The lung sections used for staining were mounted with mounting media fortified with DAPI (Vector Laboratories, Inc, Burlingame, CA, USA). To measure hydroxyproline in tissue, we used a hydroxyproline colorimetric assay kit (BioVision, Milpitas, CA, USA) per the manufacturer’s protocol.

***Cell culture***

To evaluate the role of IL-32γ in fibroblasts, the human lung fibroblast cell line MRC-5 was purchased from the American Type Culture Collection (ATCC, Manassas, VA, USA). In addition, MEF cells and primary lung fibroblasts from IL-32γ TG mice were used. These cells were cultured in MEM (Welgene, Seoul, Korea) with 10% fetal bovine serum (Gibco, Grand Island, NY, USA) and 1% penicillin-streptomycin (Welgene). Cells were seeded at a density of 2 × 10^5^ cells/well in 60-mm culture dishes and then stimulated with 5 ng/mL of TGF-β (R&D Systems, Minneapolis, MN, USA), 10 ng/mL of TNF-α (R&D Systems), 1 μg/mL of LPS (Sigma), 10 μg/mL of Poly I: C (San Diego, CA, USA), 10 ng/mL of IL-1β (R&D Systems), 50–200 µg RGD peptide (tripeptide Arg-Gly-Asp, R&D Systems), and 150 ng/mL of rIL-32 (YBDY, Seoul, Korea).

To silence intracellular IL-32γ expression, small interfering siRNA (siRNA) to IL-32γ (antisense sequence: 5′-UCAUCAGAGAGGA CCUUCGUU-3′) was used.

***Cell adhesion assay***

Crystal violet-stained cells were dissolved using 33% acetic acid and optical density values were measured at a wavelength of 550 nm. For measuring spindle-shaped cells, cells were observed and counted at 30 min after staining under a microscope (magnification: 100×).

***Western blotting***

Cells were lysed on ice in lysis buffer (Cell Signaling, Inc.) containing protease inhibitors for 30 min and then centrifuged at 18,000 ×*g* for 20 min at 4°C. Proteins were detected with antibodies against fibronectin, smad3, p-Smad3, TGF-β receptor1, JNK, p-JNK, p38, p-p38, Erk, p-Erk, paxillin, p-paxillin (all from Cell Signaling, Inc.), smurf2 and FAK (Santa Cruz Biotechnology, Dallas, TX, USA), α-SMA (Abcam, Cambridge, UK), β-actin (Bioworld, St. Louis Park, MN, USA), IL-32 (Ybdy), and p-FAK (Invitrogen, Carlsbad, CA, USA). Anti-rabbit, -goat, and -mouse secondary antibodies were purchased from Bethyl Laboratories (Montgomery, AL, USA). Protein bands were detected with ECL solution (Genedepot, Barker, TX, USA). The quantification graphs are drawn from intensity measurements using the Image J program (NIH, Bethesda, MD, USA).

***RT-PCR and semi-quantitative PCR***

Total RNA was extracted from cells using Trizol reagent (Invitrogen) and purified using RNeasy Mini Kits (Qiagen, Hilden, Germany) according to the manufacturers’ instructions. Purified RNA (1 μg) was reverse-transcribed to cDNA using oligo(dT) primers and reverse transcriptase (Roche Applied Science, Mannheim, Germany). Target amplification was performed with following primers: IL-32 sense, GACAGTGGCGGCTTATTATGAG; IL-32 antisense, CCTCGGCACCGTAATCCA T; TNF-α sense, CGCTCTTCTGCCTGCTGCACTT; TNF-α antisense, AGGCTTGTCACTCGGGGTTCGA; GAPDH sense, TGCACCACCAACTGCTTA; GAPDH antisense, GGCATGGACTGTGGTCAT; integrin α2sense, GGAACGGGACTTTCGCAT; integrin α2 antisense, GGTACTTCGGCTTTCTCATCA; integrin αv sense, AATCTTCCAATTGAGGATATCAC; integrin αv antisense, AAAACAGCCAGTAGCAACAAT; integrin β1sense, CGATGCCATCATGCAAGT; integrin β1 antisense, ACACCAGCAGCCGTGTAAC; integrin β3 sense, CCGTGACGAGATTGAGTCA; integrin β3 antisense, AGGATGGACTTTCCACTAGAA; integrin β5 sense, GGAGCCAGAGTGTGGAAACA; integrin β5 antisense, GAAACTTTGCAAACTCCCTC; integrin β8 sense, AATTTGGTAGTGGAAGCCTATC; integrin β8 antisense, GTCACGTTTCTGCATCCTTC

***His pull-down assay and immunoprecipitation***

His-tagged IL-32γ (100 µg) was incubated with Ni-NTA agarose (Qiagen) at 4°C for 2 h. The beads were then washed 3 times with buffer containing imidazole (Sigma). The supernatant of TGF-β-stimulated MRC-5 lysate was incubated with IL-32 pre-bound Ni-NTA Agarose at 4°C for 2 h. Next, proteins associated with the beads were subjected to immunoblot analysis using anti-integrin β3, anti-paxillin, or anti-His. For immunoprecipitation, flag-tagged IL-32γ-overexpressing MRC-5 cells were lysed on ice in lysis buffer (20 mM Tris, pH 7.5, 150 mM NaCl, 0.25% NP-40, 1.5 mM MgCl_2_, 1 mM phenylmethylsulfonyl fluoride). Anti-flag bound to protein G sepharose (GE Healthcare, Little Chalfont, UK) was incubated with lysates at 4 for 3 h. Beads were washed 3 times with lysis buffer. Proteins were electrotransferred to polyvinylidene fluoride membranes (GE Healthcare) and subjected to immunoblotting.
