# Supplementary figures and images for: IL-32γ attenuates airway fibrosis by modulating the integrin-FAK signaling pathway in fibroblasts
Source: Respir Res. 2018 Sep 26;19:188. doi: 10.1186/s12931-018-0863-3 (PMC6158920; doi:10.1186/s12931-018-0863-3)

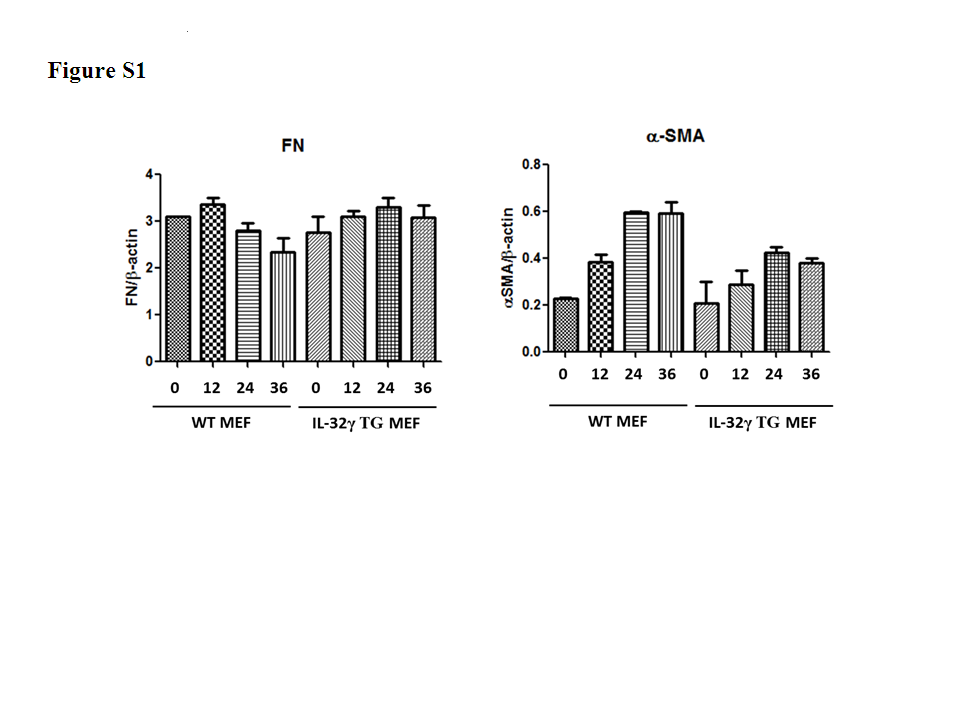

Supplement: Supplementary file 4 — Figure S1. Extracelular IL-32γ suppresses fibroblast activation. Endogenous IL-32γ did not significantly suppress the expression of fibronectin and α-SMA. (TIF 202 kb) [file 12931_2018_863_MOESM2_ESM.tif]

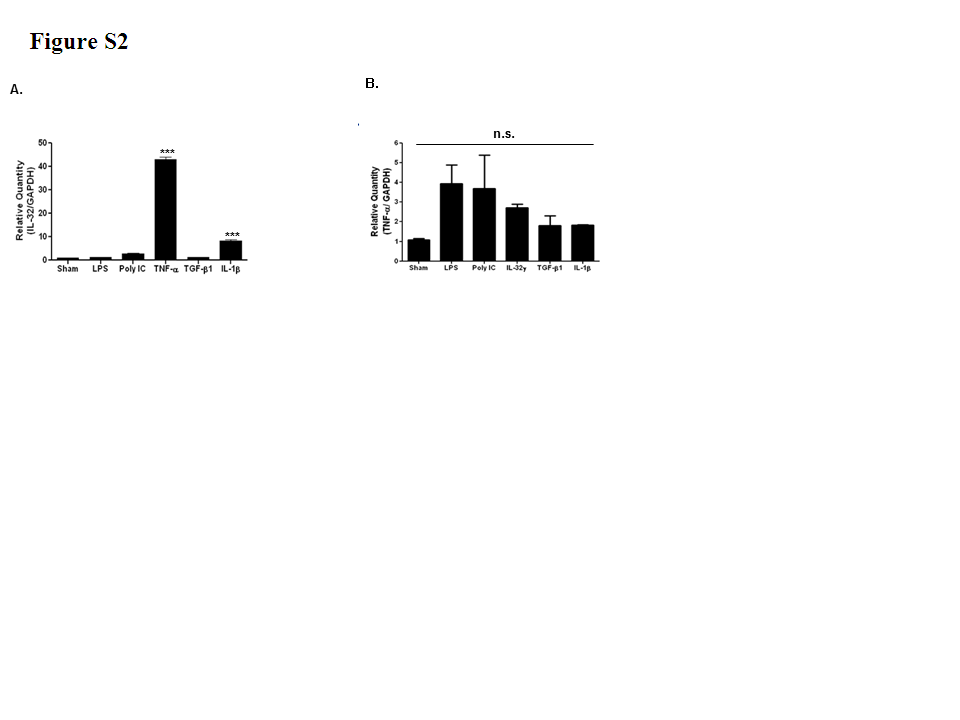

Supplement: Supplementary file 5 — Figure S2. IL-32γ mRNA expression was induced by TNF-α. MRC-5 cells were stimulated with each cytokine including LPS (1 μg/mL), Poly I: C (10 μg/mL), TNF-α (10 ng/mL), IL-32γ (150 ng/mL), TGF-β (5 ng/mL), and IL-1β (10 ng/mL). After 24-h stimulation, mRNA level of IL-32γ (A) and TNF-α (B) were measured by quantitative PCR. (TIF 71 kb) [file 12931_2018_863_MOESM3_ESM.tif]

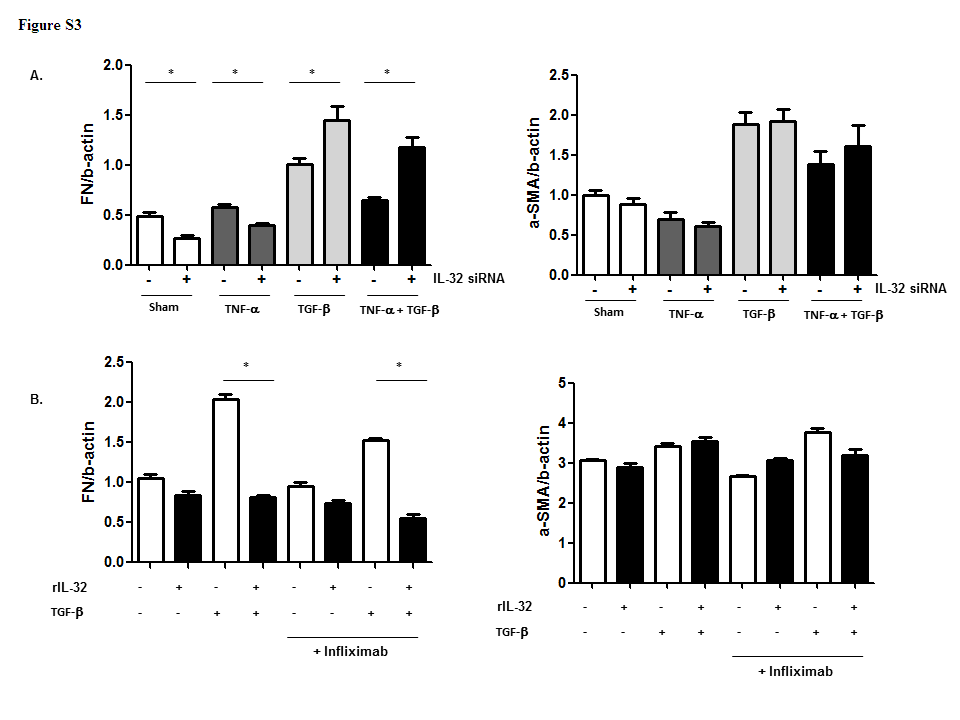

Supplement: Supplementary file 6 — Figure S3. Anti-fibrotic effect of rIL-32γ is independent of TNF-α. Anti-fibrotic effect of TNF-α was not observed in IL-32γ-knockdown MRC-5 cells (A). rIL-32γ suppressed the expression of fibronectin and α-SMA after TNF-α inhibitor treatment (B). (TIF 96 kb) [file 12931_2018_863_MOESM4_ESM.tif]

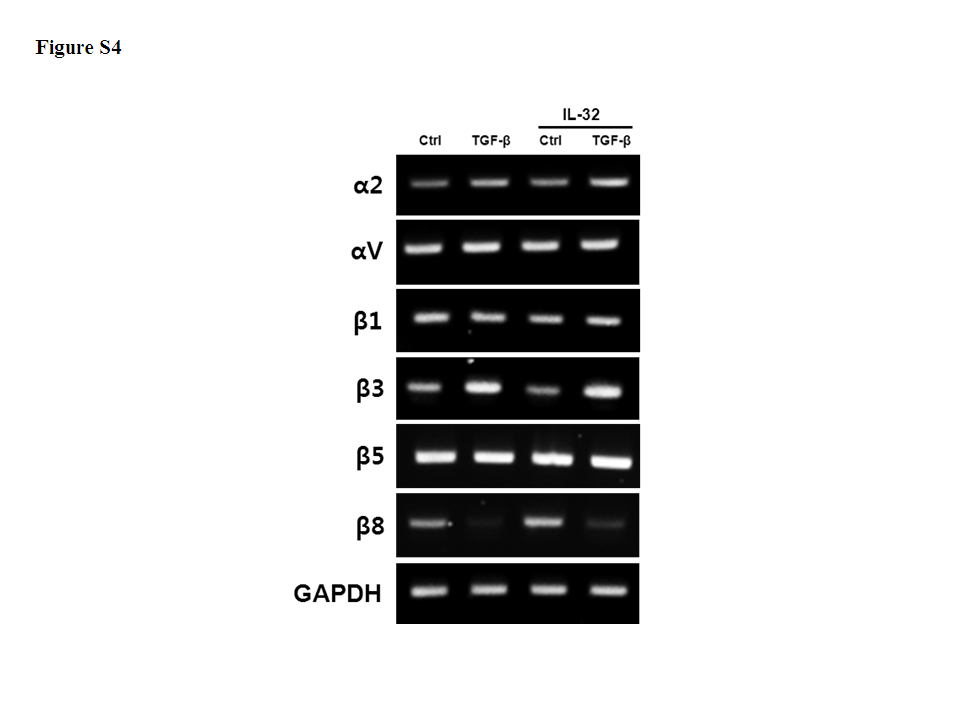

Supplement: Supplementary file 7 — Figure S4. Integrin expression in activated fibroblast is not affected by rIL-32γ. The integrin mRNA levels of α2, αv, β1, β3, β5, β8, and the GAPDH mRNA level were determined by semi-quantitative PCR in MRC-5 after TGF-β or rIL-32γ treatments. (TIF 111 kb) [file 12931_2018_863_MOESM7_ESM.tif]
